# Danning tablets attenuates α-naphthylisothiocyanate-induced cholestasis by modulating the expression of transporters and metabolic enzymes

**DOI:** 10.1186/1472-6882-14-249

**Published:** 2014-07-17

**Authors:** Lili Ding, Binfeng Zhang, Changsen Zhan, Li Yang, Zhengtao Wang

**Affiliations:** 1 The Ministry of Education (MOE) Key Laboratory for Standardization of Chinese Medicines, Institute of Traditional Chinese Materia Medica, Shanghai University of Traditional Chinese Medicine, Shanghai 201203, China; 2 Shanghai R&D Center for Standardization of Traditional Chinese Medicines, Shanghai 201203, China; 3 Shanghai Hutchison Pharmaceuticals, Shanghai 201203, China

**Keywords:** Danning tablet, α-naphthylisothiocyanate (ANIT), Cholestasis, Transporter, Metabolic enzyme

## Abstract

**Background:**

The Danning tablets (DNts) is commonly prescribed in China as a cholagogic formula. Our previous studies showed that DNts exerted the protective effect on α-naphthylisothiocyanate (ANIT)-induced liver injury with cholestasis in a dose-dependent mannar. However, the detailed molecular mechanisms of DNts against ANIT-induced cholestasis are still not fully explored.

**Methods:**

Danning tablet (3 g/kg body weight/day) was administered orally to experimental rats for seven days before they were treated with ANIT (60 mg/kg daily via gastrogavage) which caused cholestasis. Serum levels of alanine aminotransferase (ALT), aspartate aminotransferase (AST), alkaline phosphatase (ALP), total bilirubin (T-Bil), direct bilirubin (D-Bil) and total bile acid (TBA) were measured to evaluate the protective effect of Danning tablet at 12, 24 and 48h after ANIT treatment. Meanwhile, total bilirubin or total bile acid in the bile, urine and liver were also measured at 48h after ANIT treatment. Furthermore, the hepatic or renal mRNA and protein levels of metabolic enzymes and transports were investigated to elucidate the protective mechanisms of Danning tablet against ANIT-induced cholestasis.

**Results:**

In this study, we found that DNts significantly attenuated translocation of multidrug resistance-associated protein 2 (Mrp2) from the canalicular membrane into an intracellular and up-regulated the hepatic mRNA and protein expressions of metabolic enzymes including cytochrome P450 2b1(Cyp2b1) and uridine diphosphate-5¢- glucuronosyltransferase (Ugt1a1)) and transporters including bile salt export pump (Bsep) and multidrug resistance protein 2 (Mdr2)) as well as renal organic solute transporter beta (Ostβ), accompanied by further increase in urinary and biliary excretion of bile acid and bilirubin.

**Conclusions:**

DNts might promote bile acid and bilirubin elimination by regulating the expressions of hepatic and renal transporters as well as hepatic metabolic enzymes.

## Background

Cholestasis, an impairment or cessation in the flow of bile, leads to hepatic and systemic accumulation of potentially toxic biliary compounds such as bile acids and bilirubin, resulting in acute liver toxicity, jaundice and hypercholesterolemia, and then aggravated outcomes just like hepatic fibrosis, cirrhosis and clinical signs of liver failure [1,2]. It has been reported that cholestasis may develop from viral hepatitis or the administration of certain drugs and hormones or mechanical obstructions in the extrahepatic bile ducts [2].

The mechanisms of cholestasis might be related to the altered expressions of hepatic metabolic enzymes and transporters [3,4]. Drug metabolic enzymes (DMEs) play central roles in the metabolism, elimination and/or detoxification of xenobiotics or exogenous compounds introduced into the body [5]. Metabolic enzymes in the liver are classified into phase 1, such as cytochrome P450 (Cyp) [5], and phase 2, such as uridine diphosphate-5¢- glucuronosyltransferase (Ugt) [6], sulfotransferase (Sult) [7]. Several transporters which are related to cholestasis have been identified in hepatocytes, such as multidrug resistance protein (Mdr), bile salt export pump (Bsep), multidrug resistance associated protein (Mrp) [8,9], organic solute transporter alpha/beta (Ostα/β) [10], Na^+^ taurocholate cotransporting polypeptide (Ntcp) [9], and organic anion transporting polypeptide (Oatp) [11].

ANIT is well konwn as a cholestatic compound and has been widely used as a model component for induction of intrahepatic cholestasis [12]. In rodents, α-naphthylisothiocyanate (ANIT) is a widely used chemical to mimic human intrahepatic cholestasis [13]. It has been reported that the mRNA expressions of metabolic enzymes and transporters changed in ANIT-induced cholestasis model [14,15].

Danning tablets (DNts), as a composite prescription of traditional Chinese medicine, consisting of seven medicinal materials: *Radix et Rhizoma Rhei, Rhizoma et Radix Polygoni Cuspidati, Pericarpium Citri Reticulatae, Pericarpium Citri Reticulatae Viride, Radix Curcumae, Fructus Crataegi and Rhizoma Imperatae*[16], have been proved to be effective for treatment of cholecystitis and prevention the formation of cholesterol gallstone in human [17]. We previously showed that the protective effect of DNts on ANIT-induced liver injury was probably due to its attenuation of oxidative stress disruption in the liver tissues and neutrophil infiltration [18]. However, the underlying mechanisms about protective effect of DNts on ANIT-induced cholestasis are not explored yet.

In this study, in order to clarify the possible mechanisms for protective effect of DNts on ANIT-induced cholestasis, we investigated the mRNA and protein expressions of metabolic enzymes and transports which were relevant to the excretion of bile acids and bilitubin.

## Methods

### Drugs, chemicals and reagents

DNts were friendly provided by Shanghai Hutchison Pharmaceuticals (Shanghai, China) at July 2010. Standard compounds (emodin, aleo-emodin, rhein, chrysophanol, physcion) purchased from National Institute for the Control of Pharmaceutical and Biological Products (Beijing, China). ANIT were purchased from Sigma-Aldrich (St. Louis, USA). The purity of each compound was determined to be above 98% by HPLC analysis. Ammonium acetate, formic acid, acetonitrile and methanol (HPLC-grade) were purchased from Fisher Scientific (Nepean, Ont., Canada). Ultrapure water was prepared by a Milli-Q50 SP Reagent Water System (Millipore Corporation, MA, USA) for the preparation of samples and buffer solutions. Serum levels of ALT, AST, ALP, T-Bil, D-Bil and TBA were determined using a commercially available test kits (SHINO-TEST Corporation, Japan) with HITACHI 7080 Automatic Analyzer system (Japan). All other chemicals and solvents used were of analytical grade.

### Chromatography analysis

In order to make quality control of DNts and improve the repeatability of experiments, the contents of five anthraquinones, which might be the main active components contained in DNts, were determined by HPLC analysis. The determination was performed on the Agilent 1100 chromatographic system, consisting of a solvent degasser, aquaternary gradient pump before column, diodearray detector (DAD) and a data station with analytical software of Chemstation 8.03 (Agilent, Inc., USA). The method to analyze the anthraquinones in DNts used in this study was well established according to the China Pharmacopoeia Committee (2010 edition) [15]. All the separation was performed on a Kromasil® C18 analytical column (250 mm × 4.6 mm, 5 μm) and maintained at 25°C. The mobile phase was a mixture of methanol–0.1% phosphoric acid aqueous solution (85:15, v:v) with the flow rate of 1ml/min. The injection volume was 10 μl and the detection wavelength was set at 254 nm. The standard solutions of five AQs (aloe-emodin 15 μg/mL, emodin 8 μg/mL, chrysophanol 40 μg/mL, rhein 2 μg/mL and physcion 10 μg/mL) were prepared with methanol. Samples were prepared according to China Pharmacopoeia Committee, 2010.

### Animal experimental design

Male Wistar rats weighing 220 ± 20 g were used in this study. All rats were maintained in 12-h light/dark cycles and given free access to water and standard chow. All animal care and use procedures were in accordance with the guidelines of the Institutional Animal Care and Use Committee and were approved by the Animal Experimentation Ethics Committee at the Shanghai University of Traditional Chinese Medicine under the guidelines of the National Health and Medical Research Council of China. The animals were randomly divided into the following treatment groups (n = 8 rats/group): (1) Control group (Vehicle group), (2) ANIT group, (3) DNts + ANIT group, (4) DNts group. The rats in Control and ANIT groups were pretreated with 0.5% sodium carboxymethylcellulose (CMCNa) solution alone; the other two groups were intragastrically treated daily with the powder of DNts which was suspended in 0.5% CMCNa solution at a dose of 3.0 g/kg B.W. for 9 consecutive days, respectively. At 12 h after the seventh administration of test drugs, groups 2 and 3 were intragastrically received ANIT dissolved in peanut oil at a dose of 60 mg/kg B.W.. The Control and DNts groups were intragastrically given the peanut oil in an equal volume as for groups 2 and 3 (5 ml/kg B.W.). Blood samples were collected from inferior vena cava at 0, 12, 24, and 48 h after ANIT administration and separated, and then stored at −80°C for later analysis of biochemical parameters.

At 48 h post ANIT administration, the rats were anaesthetized with urethane (1 g/kg B.W., i.p.). The bile duct was cannulated with Closed IV Catheter System (inner diameter, 0.36 mm; outer diameter, 0.71 mm; Becton, Dickinson and Co., Suzhou, China). After the operation, Bile specimens were collected into tubes for 1 h, as previously described [18]. Then, the animals were killed by decapitation, the liver and kidney of rats were collected and stored in liquid nitrogen for analysis of enzyme levels and gene or protein expressions.

### Serum biochemistry analysis

Serum was collected at 0, 12, 24, 36 and 48 h after ANIT treatment. The serum levels of aspartate aminotransferase (AST), alanine aminotransferase (ALT), alkaline phosphatase (ALP), total bilirubin (TBil), direct bilirubin (DBil) and total bile acid (TBA) were determined using commercially available clinical test kits and an automatic biochemical analyzer (HITACHI 7080, Japan).

### Bile acid and bilirubin analysis

Bile acids were extracted from liver samples using 75% ethanol, as previously described [19]. Total bile acids in the liver, bile, and urine were measured using commercial assay kits from the Jiancheng Institute of Biotechnology (Nanjing, China). Total bilirubin in the bile and urine were also measured with commercial assay Kits from the Jiancheng Institute of Biotechnology (Nanjing, China) in accordance with the manufacturer’s protocols.

### Quantitative real-time PCR analysis

Total RNA from liver and kidney were extracted using TRIzol reagent (Invitrogen, CA, USA) according to the manufacturer’s instructions. Quantitative real-time PCR (qPCR) was performed using cDNA generated from 3 μg total RNA with the SuperScript II Reverse Transcriptase kit (Invitrogen, CA, USA). PCR reactions were carried out using SYBR Premix ExTaq Mix (Takara Biotechnology, Japan) in an ABI Prism 7900HT Sequence Detection System (Applied Biosystems, CA). The primer sequences were listed in Table 1. Thermal cycler parameters were as follows: 1 cycle of 95°C for 30 s, 40 cycles of denaturation (95°C, 5 s) and combined annealing/extension (60°C, 30 s). Gene expression changes were calculated by the comparative Ct method and the values were normalized to endogenous reference Gapdh.

**Table 1:** Primer sequences used for quantitative real-time PCR in this study

**Gene**	**(GenBank accession no.)**	**Sequence (5’–3’)**
Asbt	XM_003751634	GGATAGATGGTGACATGGAC (fw)
		GAAACAGGAATAACAAGCGC (rev)
Bsep	U69487	CTGATGCTTATGGGAGGCGTAT (fw)
		TCTGGTGGAAGGAGCTGTTGATC (rev)
Cyp2b1	NM_001134844	GCTATGGTTTCCTGCTGATGCTC (fw)
		GACTCTGTGTGGTACTCCAATAGGG (rev)
Mdr2	NM_012690	GTTGAAATGAAAATGTTGGCTGG (fw)
		ATGCCGTAGATGTGAGCCTTCC (rev)
Mrp2	NM_012833	TCAGATGAGGAGGTTTGGAGGG (fw)
		CTCTGTCACTTCGGATAACAACCC(rev)
Mrp3	NM_080581	TGAAGAGACTGGAATCCGTTAGCC (fw)
		GTTGGAGGCGATGTAAGGATAAGTG (rev)
Mrp4	NM_133411	GACACCAAAAACGCGAACGG (fw)
		ACAAGGACATAGAACACCAGCAGG (rev)
Ntcp	NM_017047	CTACCTCCTCCCT GATGCCCTT (fw)
		TGAGGATGGTAGAACAGAGTTGAATG (rev)
Oatp1	NM_017111	TGCCTGCCTTCTTCATTCTGATACT (fw)
		GTTTTCAGTTCTCCGTCATTCTCG (rev)
Ostα	NM_001107087	TCCTCTGTCCCTGAGCCTTATC (fw) TCTCTGCTTTCACCATTTATTACAA (rev)
Ostβ	XM_238546	TGCTTTGGTATTTCCGTTC (fw)
		TCTGGCATTCCGTTGTCTT (rev)
Sult2a1	NM_131903	ATCAAAAAGAAAGGACCACGACTC (fw)
		AACCATTCAACATAAGTTCCCAGTG (rev)
Ugt1a1	NM_012683	TGAAGCCTATGTCAACGCCTC (fw)
		CGATGGTCTAGTTCCGGTGTAGC (rev)
Gapdh	NM_017008	AGAAGGTGGTGAAGCAGGCATC (fw)
		CGAAGGTGGAAGAGTGGGAGTTG (rev)

fw: forward primer; rev: reverse primer; bp: base pair.

### Preparation of liver membranes and for Western analysis

Plasma membranes or intracellular microsomal membranes were separated by differential centrifugation. Portions of the liver were homogenized with a teflon pestle (20 strokes at 3,000 rpm) in 0.3 mol/L sucrose containing 0.1 mmol/L PMSF, 25 μg/mL leupeptin, 5μg/mL aprotinin, and 5 μg/mL pepstatin A (50 mg liver/mL buffer) according previous report [20]. The homogenate was used for membrane preparation as described previously [21]. Protein concentrations were measured according to the method of BCA [22] using bovine serum albumin as a standard.

### Western blot studies

Immunoblotting and subsequent densitometry were performed with mixed plasma membranes, and intracellular membranes were incubated with antibodies against Mrp2 (ab3373, 1:50, abcam) and Na^+^/K^+^-ATPase (#3010, 1:1000, Cell Signaling). Moreover, the crude membranes were prepared according to the protocol of protein extraction kit (BioVision, USA). Equal amounts of crude membranes (40 μg/lane) were separated on 6%-15% SDS-PAGE and transferred to nitrocellulose membrane. Membranes were blocked in 5% skim milk and incubated with antibodies against Bsep (sc-25571, 1:200, Santa Cruz), Ntcp (#6522-1, 1:1000, epitomics), Oatp1 (LS-C113034-50, 1:1000, LifeSpan BioSciences), Mrp3(ab3375, 1:50, abcam), Mdr2 (SAB2100008, 1:1000, sigma) and Na^+^/K^+^-ATPase (#3010, 1:1000, Cell Signaling), respectively. Blots were incubated with horseradish peroxidase conjugated secondary antibodies (Santa Cruz) and developed by ECL detection regents (Amersham). The protein bands were quantified by the average ratios of integral optic density (IOD) following normalization to Na^+^/K^+^-ATPase expression.

### Determination of liver Ugt1a1, Cyp2b1 and Sult2a1 levels

Liver segments were homogenized in ice-cold PBS. The homogenates were centrifuged at 3,000 g for 10 min and the supernatants were assayed for the determination of levels of the Ugt1a1, Cyp2b1 (CUSABIO, CN) and Sult2a1 (USCN Life Science,CN) by using ELISA kits according to the manufacturer’s protocols. The results are expressed as folds of control.

### Statistical analysis

The data were analyzed using a SPSS 16.0 statistical package. Multiple comparisons were performed by one-way analysis of variance (ANOVA) followed by LSD t-test. Difference was considered statistically significant when *p* ≤ 0.05, and very significant when *p* ≤ 0.01 and *p* ≤ 0.001. All results are presented as the mean ± SD.

## Results

### HPLC determination of anthraquinones in DNts

**Figure 1:**
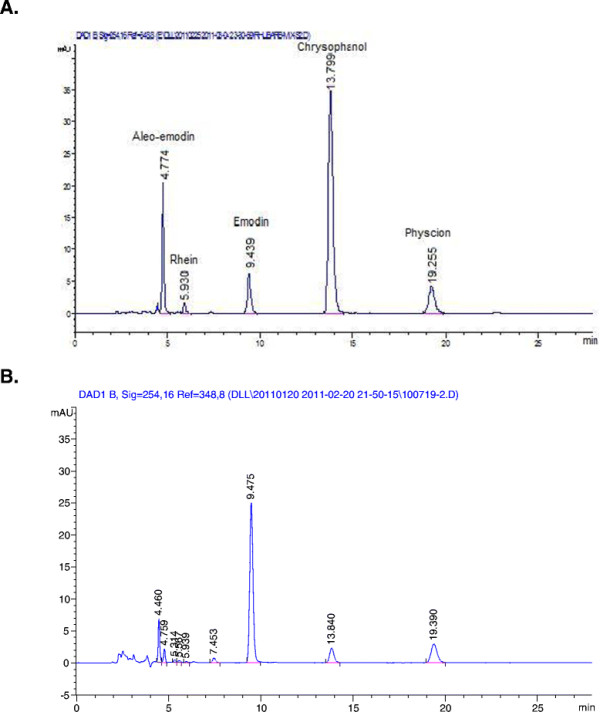
HPLC chromatogram of mixed standards (A) and DNts (B).

The major compounds in DNts were anthraquinones and analyzed with HPLC- UV. By referring to standards, HPLC chromatogram analysis of three batches of DNts showed that the main anthraquinones in DNts were aloe-emodin, emodin, chrysophanol and physcion (Figure 1); the contents of these components were 0.445 mg/g, 13.452 mg/g, 1.341 mg/g, and 2.290 mg/g, respectively, and summarized in Table 2.

**Table 2:** The contents of five anthraquinones in DNts

**Compound**	**RT**	**Content (mg/g)**	**SD**
Aleo-emodin	4.774	0.445	0.035
Emodin	9.439	13.452	0.843
Chrysophanol	13.799	1.341	0.094
Physcion	19.255	2.290	0.168
Rhein	-	-	-

RT:retention time.

### Effects of DNts on the levels of serum enzymes and components in ANIT-induced cholestasis rats

**Figure 2:**
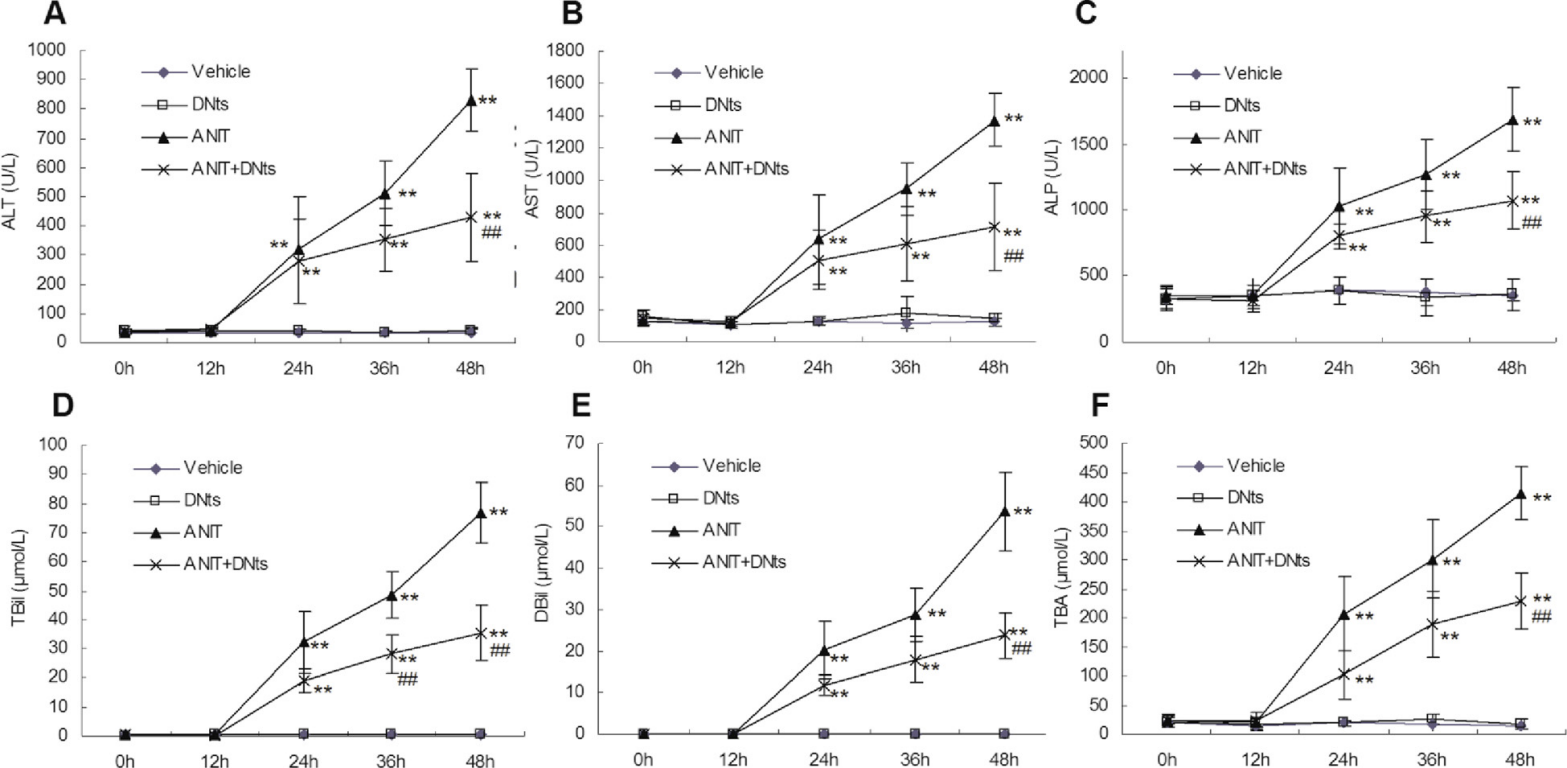
**Serum levels of (A) ALT, (B) AST, (C) ALP, (D) T-Bil, (E) D-Bil and (F) TBA were measured in each group at 0, 12, 24, 36 and 48 h after ANIT treatment.** Data represent means ± S.D. (n = 8 for each group). Significant difference between two groups. *p < 0.05, **p < 0.01 vs. control group; ^#^*p* < 0.05, ^##^*p* < 0.01 vs. the ANIT-treated group.

To examine the effects of DNts on ANIT mediated hepatotoxicity and cholestasis, plasma levels of ALT, AST, ALP, TBil, DBil and TBA were quantified as measures of liver injury with cholestasis. In both ANIT group and DNts + ANIT group, serum levels of ALT, AST, ALP, TBIL, DBIL and TBA were unchanged at 12 h, but significantly elevated at 24 h and 36h and peaked at 48 h after ANIT treatment as compared to those in the vehicle group. However, the pretreatment of DNts at dose of 3 g/kg B.W. significantly reversed ANIT-induced elevation of TBIL (Figure 2; *p* < 0.01) at 36 h as well as ALT, AST, ALP, TBIL, DBIL and TBA (Figure 2; *p* < 0.01) at 48 h after ANIT treatment. In addition, DNts treated alone (3 g/kg B.W.) did not significantly affect serum ALT, AST, ALP, TBil, DBil and TBA levels as compared to the vehicle group (Figure 2). The above results are consistent with our previous report [18].

### Effects of DNts on the levels of bile acids and bilirubin in the liver, urine, and bile after ANIT treatment

**Figure 3:**
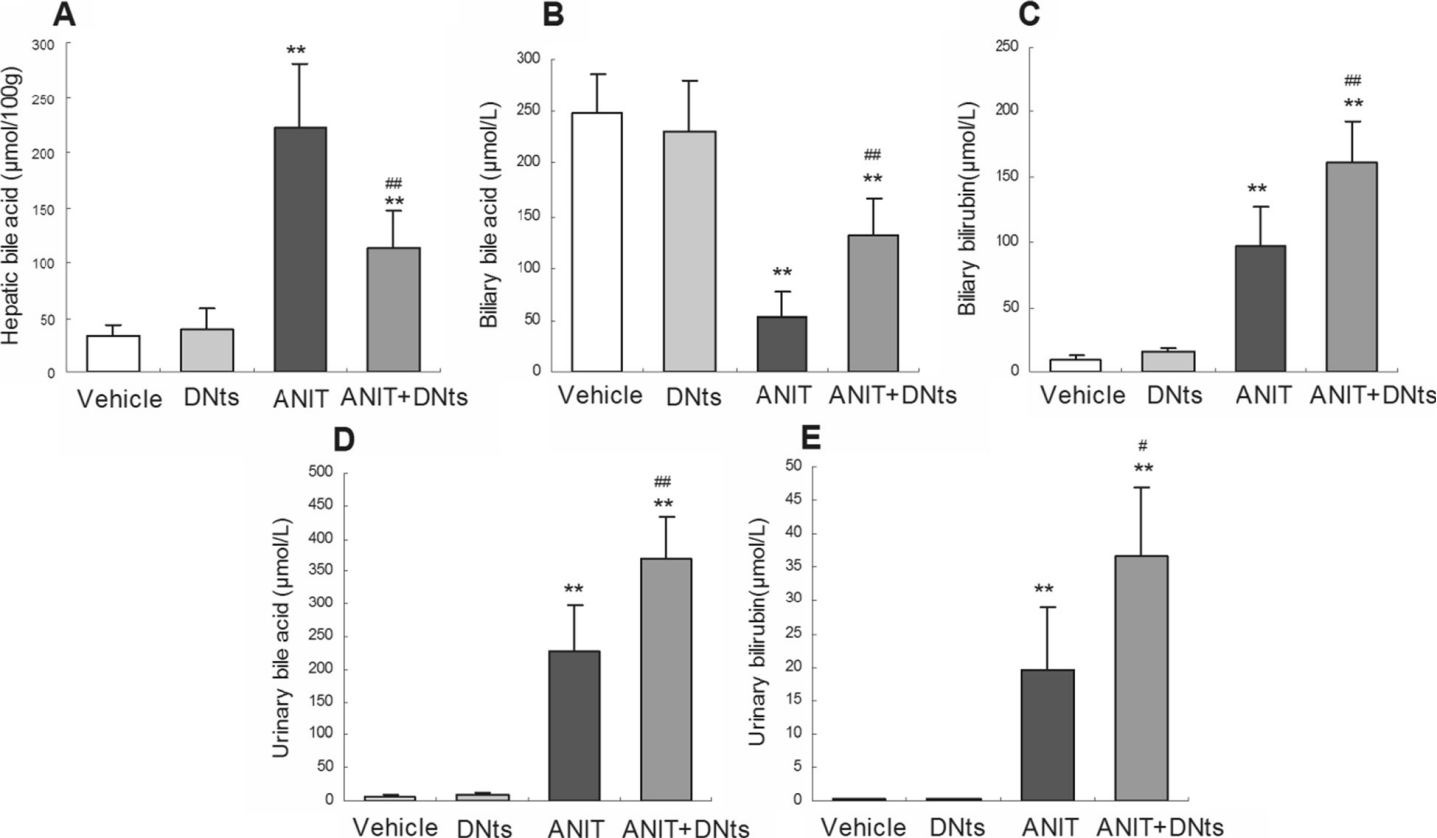
**Altered levels of bile acids and bilirubin after ANIT treatment. (A)** hepatic bile acids, **(B)** biliary bile acids, **(C)** biliary bilirubin, **(D)** urinary bile acids, and **(E)** urinary bilirubin were measured in each group 48 h after ANIT treatment. Data represent means ± S.D. (n = 8 for each group). Significant difference between two groups. *p < 0.05, **p < 0.01 vs. control group; ^#^*p* < 0.05, ^##^*p* < 0.01 vs. the ANIT-treated group.

The concentrations of bile acid or bilirubin in the liver, bile, and urine were quantified at 48 h after ANIT treatment. Hepatic bile acid pool size were increased significantly in ANIT-treated rats (Figure 3A; *p* < 0.01). Meanwhile, urinary excretion of bile acids and bilirubin in the rats of ANIT-treated group were also increased after ANIT treatment (Figure 3D, 3E; *p* < 0.01). In addition, biliary bilirubin concentrations in ANIT-treated rats were elevated significantly (Figure 3C; *p* < 0.01). In contrast, biliary bile acid pool size were dramatically reduced by ANIT-treatment (Figure 3B; *p* < 0.01). DNts pretreatment significantly attenuated the ANIT-induced accumulation of hepatic bile acids and significantly increased the bile acid and bilirubin excretion of urine and bile in ANIT-intoxicated rats (Figure 3A- 3E; *p* < 0.01, *p* < 0.05). DNts treated alone (3 g/kg B.W.) did not significantly affect the bile acid and bilirubin pool size in the liver, bile and urine as compared to the vehicle group (Figure 3). These findings suggested that DNts pretreatment stimulates bile acid and bilirubin elimination to attenuate cholestasis.

### Effects of DNts on gene expressions of transporter and metabolic enzyme in ANIT-induced cholestasis rats

**Figure 4:**
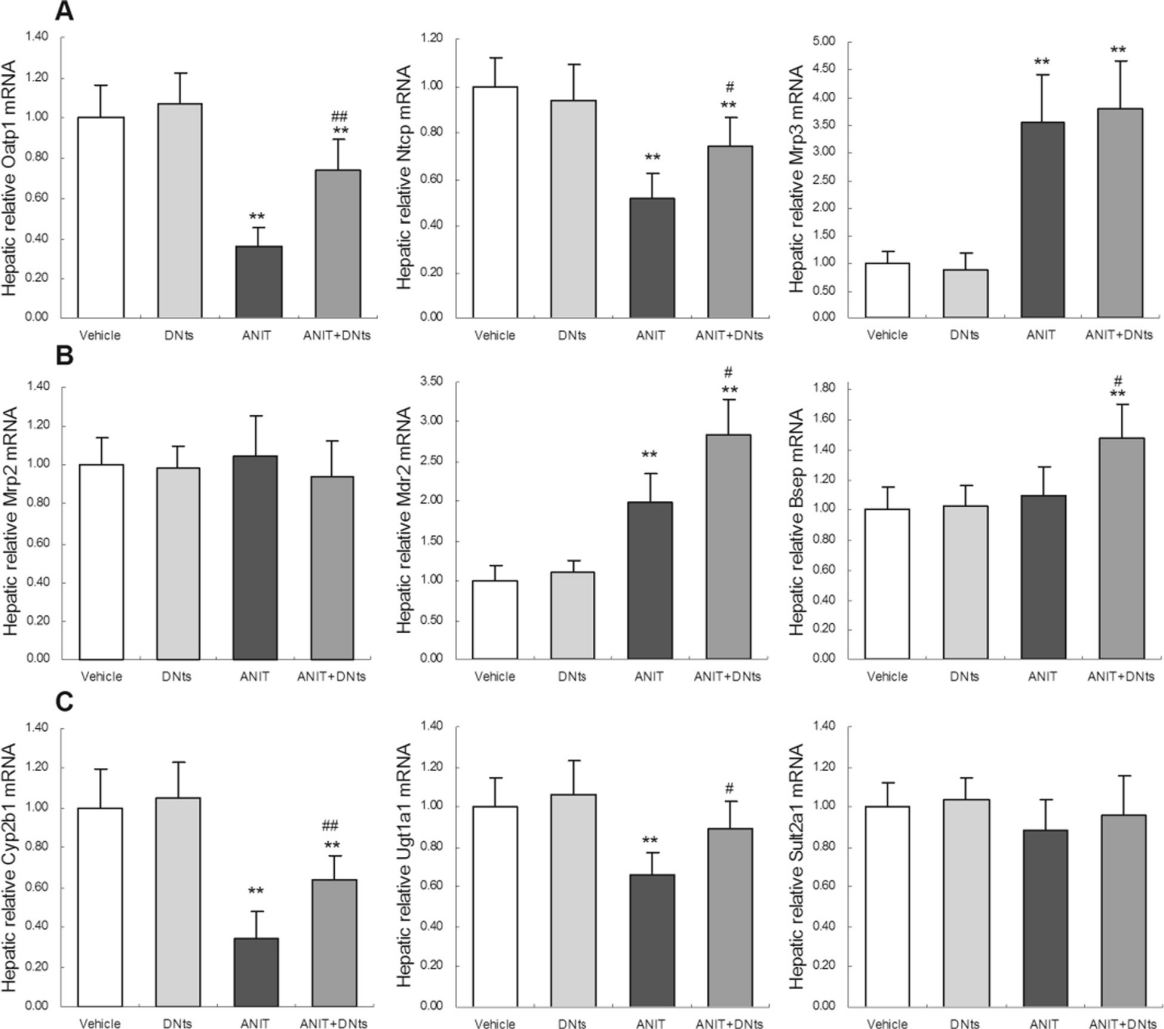
**The effects of DNts on mRNA expressions of transporters and metabolic enzymes in the liver of ANIT-induced cholestasis rats.** Relative hepatic mRNA levels of **(A)** basolateral uptake transporters (Oatp1, Ntcp) and basolateral efflux transporters (Mrp3), **(B)** basolateral efflux transporters (Mrp4, Ostα and Ostβ) **(C)** canalicular efflux transporters (Mrp2, Mdr2 and Bsep), and **(D)** bile acid-metabolizing enzymes (Cyp2b1, Ugt1a1 and Sult2a1) were determined by qRT-PCR in liver samples isolated from rats treated with vehicle, DNts, ANIT or DNts + ANIT (n = 3 for each group). Expression was normalized to Gapdh, and each bar represents the mean ± SD of three independent experiments. Significant difference between two groups. *p < 0.05, **p < 0.01 vs. control group; ^#^*p* < 0.05, ^##^*p* < 0.01 vs. the ANIT-treated group.

Firstly, we examined hepatic mRNA expressions of basolateral transporters after ANIT treatment (Figure 4A). In agreement with previous observations [14,15], the mRNA levels of basolateral uptake transporters Ntcp and Oatp1 were significantly down-regulated by ANIT (Figure 4A; *p* < 0.01). In contrast, the mRNA levels of basolateral efflux transporter Mrp3, MRP4, Ostβ were markedly up-regulated in ANIT-intoxicated rats (Figure 4A, 4B; *p* < 0.01). However, Both ANIT and DNts did not affect the mRNA expressions of Ostα (Figure 4B). DNts pretreatment significantly reversed ANIT-induced down-regulation of the Ntcp (*p* < 0.05) and Oatp1 mRNA levels (*p* < 0.01), but did not affect the mRNA levels of Mrp3, Mrp4 and Ostβ in ANIT- intoxicated rats (Figure 4A, 4B).

Secondly, hepatic gene expressions of canalicular efflux transporters were determined (Figure 4B). The mRNA level of Mdr2 was significantly up-regulated by ANIT (Figure 4C; *p* < 0.01). There were no changes in mRNA levels for Mrp2 and Bsep after ANIT treatment (Figure 4C). DNts pretreatment resulted in substantial elevation of Mdr2 and Bsep mRNA levels in ANIT-intoxicated rats (Figure 4C; *p* < 0.05).

Thirdly, we examined the hepatic gene expressions of metabolic enzymes such as Cyp2b1, Ugt1a1 and Sult2a1 (Figure 4D). The mRNA levels of Cyp2b1 and Ugt1a1 in ANIT-treated rats were significantly decreased (Figure 4D; *p* < 0.01). DNts pretreatment significantly reversed ANIT- induced reduction of the Cyp2b1 and Ugt1a1 mRNA levels (Figure 4D; *p* < 0.05), but did not affect the mRNA expression of Sult2a1 in ANIT-intoxicated rats (Figure 4D). Otherwise, DNts pretreatment alone did not affect mRNA levels of Oatp1, Ntcp, Mrp3, Bsep, Mdr2, Mrp2, Cyp2b1, Ugt1a1 and Sult2a1 (Figure 4D).

**Figure 5:**
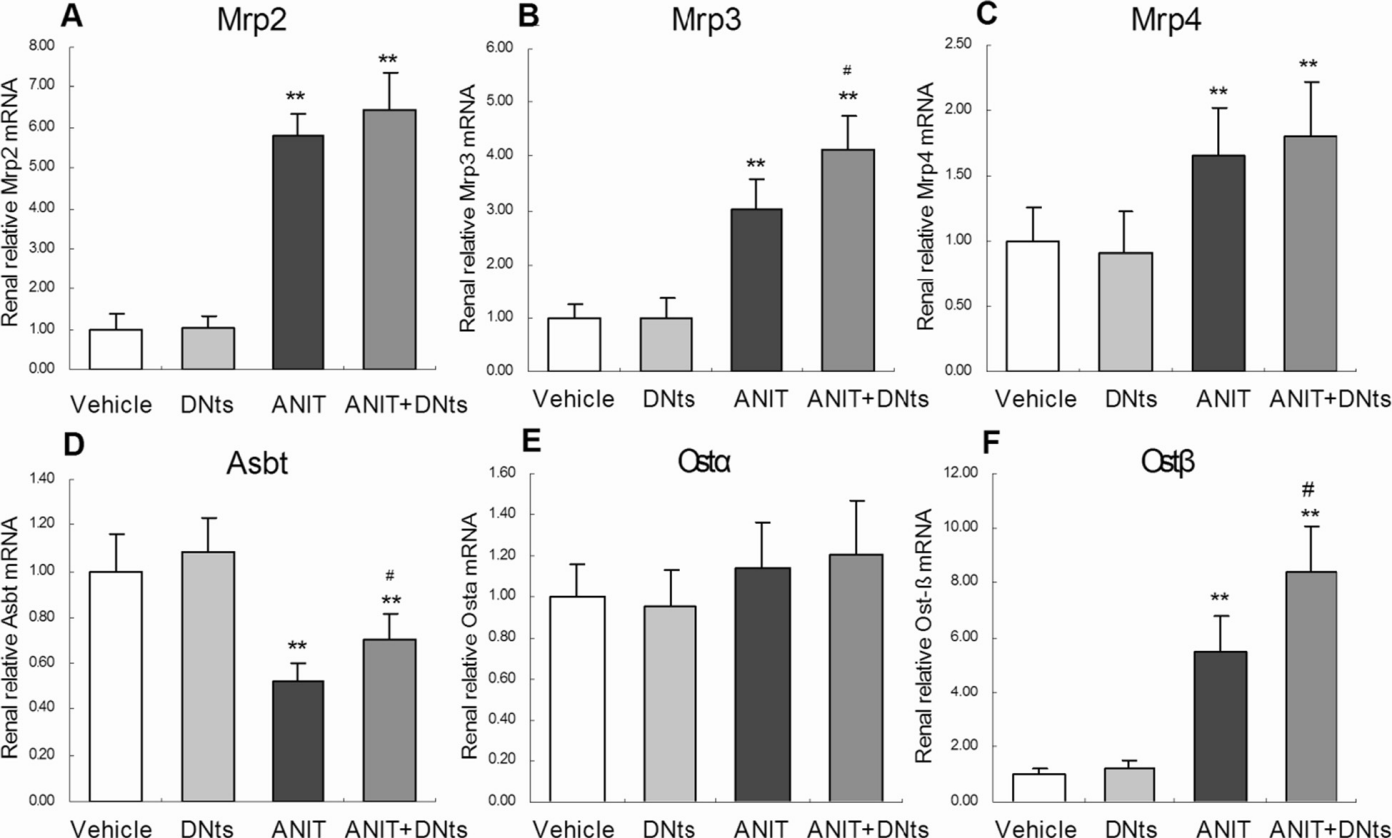
**The effects of DNts on mRNA expressions of renal transporters (A) Mrp2, (B) Mrp3, (C) Mrp4, (D) Asbt, (E) Ostα and (F) Ostβ in ANIT-induced cholestasis rats were determined by qRT-PCR (n = 3 for each group).** Expression was normalized to Gapdh, and each bar represents the mean ± SD of three independent experiments. Significant difference between two groups. *p < 0.05, **p < 0.01 vs. control group; ^#^*p* < 0.05, ^##^*p* < 0.01 vs. the ANIT-treated group.

In addition, as urinary excretion of bile acids and bilirubin were significantly higher in ANIT-intoxicated rats, we next determined the expression of renal bile acid transporters (Figure 5A-F). ANIT treatment markedly increased the mRNA levels for bile acid export transporter Mrp2, Mrp3, MRP4 and Ostβ (Figure 5A, 5B, 5C and 5F; *p* < 0.01). In contrast, the main reuptake transporter Asbt was down-regulated after ANIT treatment (Figure 5D; *p* < 0.01). Pretreatment with DNts resulted in further significantly increasing of renal Mrp3 and Ostβ mRNA levels and significantly reversing the renal mRNA level of Asbt in ANIT-intoxicated rats (Figure 5B,F; *p* < 0.05). Both ANIT and DNts treatment did not affect the mRNA expression of Ostα (Figure 5E). DNts pretreatment alone did not affect the mRNA levels of renal Mrp2, Mrp3, MRP4, Asbt, Ostα and Ostα (Figure 5A-F).

### Effect of DNts on protein expressions of Ntcp, Oatp1, Mrp3, Mdr2, Bsep and Mrp2 in the liver membranes of ANIT-induced cholestasis rats

**Figure 6:**
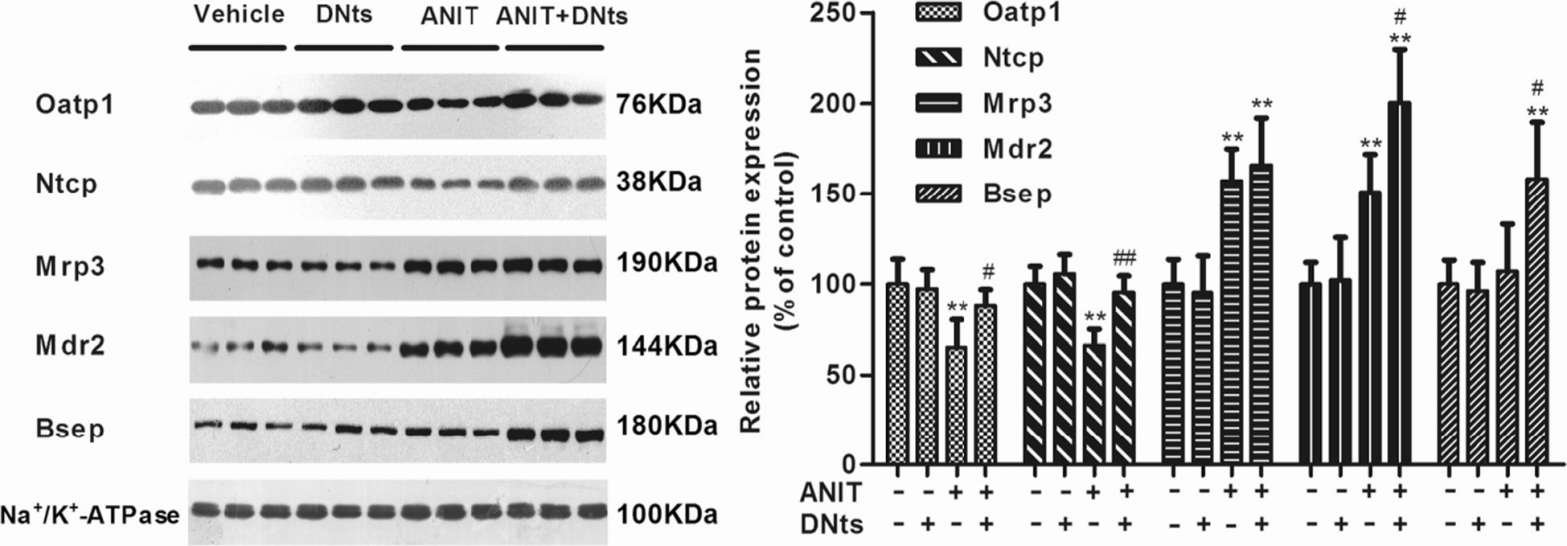
**The hepatic protein levels of transporters Oatp1, Ntcp, Mrp3, Mdr2 and Bsep were determined by Western blot (n = 3 for each group).** Quantification of the protein expressions was performed by densitometric analysis of the blots following normalization to Na^+^/K^+^-ATPase expression. Each bar represents the mean ± SD of three independent experiments. Significant difference between two groups. *p < 0.05, **p < 0.01 vs. control group; ^#^*p* < 0.05, ^##^*p* < 0.01 vs. the ANIT-treated group.

**Figure 7:**
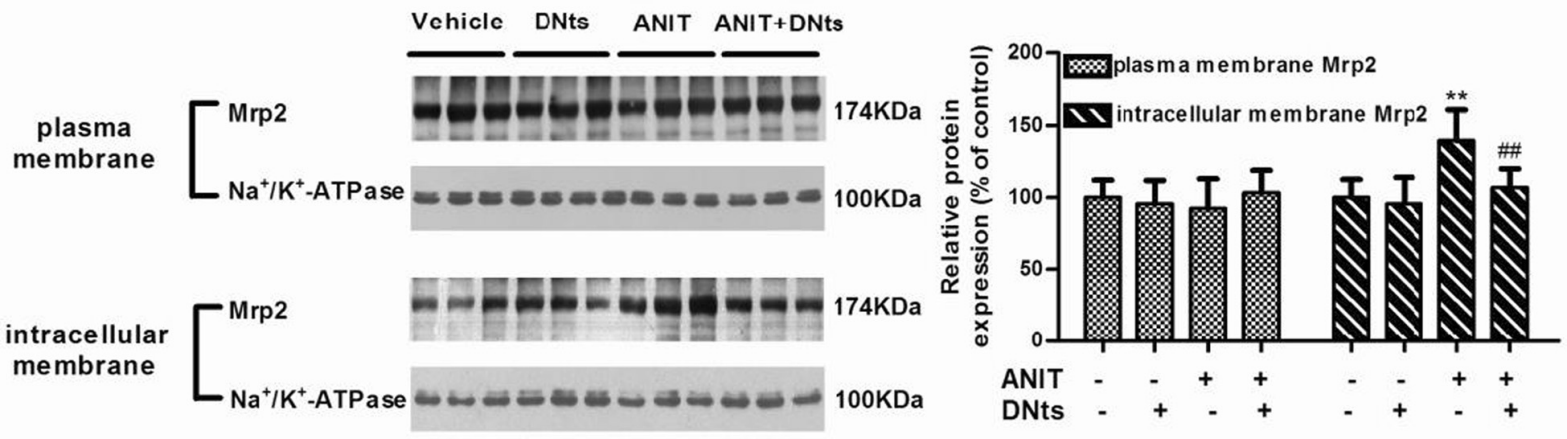
**Effects of DNts on hepatic protein levels of Mrp2 in mixed plasma membranes and intracellular membranes were determined by Western blot (n = 3 for each group).** Quantification of the protein expressions was performed by densitometric analysis of the blots following normalization to Na^+^/K^+^-ATPase expression. Each bar represents the mean ± SD of three independent experiments. Significant difference between two groups. *p < 0.05, **p < 0.01 vs. control group; ^#^*p* < 0.05, ^##^*p* < 0.01 vs. the ANIT-treated group.

Furthermore, we investigated the protein expressions of hepatic transporters to confirm the results of mRNA levels. The protein levels of basolateral uptake transporter Ntcp and Oatp1 were significantly downregulated in the liver membranes of ANIT-intoxicated rats (Figure 6; *p* < 0.01). In contrast, the protein expression of basolateral efflux transporter MRP3 was significantly up-regulated in the liver membranes of ANIT-intoxicated rats as well as the canalicular efflux transporter Mdr2 (Figure 6; *p* < 0.01). However, DNts pretreatment significantly up-regulated the protein levels of Ntcp and Oatp1 (Figure 6; *p* < 0.01, *p* < 0.05) and further increased the protein levels of canalicular efflux transporter Mdr2 and Bsep (Figure 6; *p* < 0.05), but did not affect the protein level of Mrp3 in the hepatic membranes of ANIT-intoxicated rats. Otherwise, both ANIT and DNts treatment did not affect the protein level of Mrp2 in hepatic plasma Membranes (Figure 7). Therefore, we evaluated whether ANIT could lead to redistribution of the protein Mrp2 from plasma to intracellular membranes. Mrp2 detection was increased in hepatic intracellular membranes of ANIT-intoxicated rats (Figure 7; *p* < 0.01). DNts pretreatment significantly reversed ANIT-induced up-regulation of Mrp2 in intracellular membranes (Figure 7; *p* < 0.01).

### Effect of DNts on the protein levels of Cyp2b1, Ugt1a1 and Sult2a1 in the liver of ANIT-induced cholestasis rats

**Figure 8:**
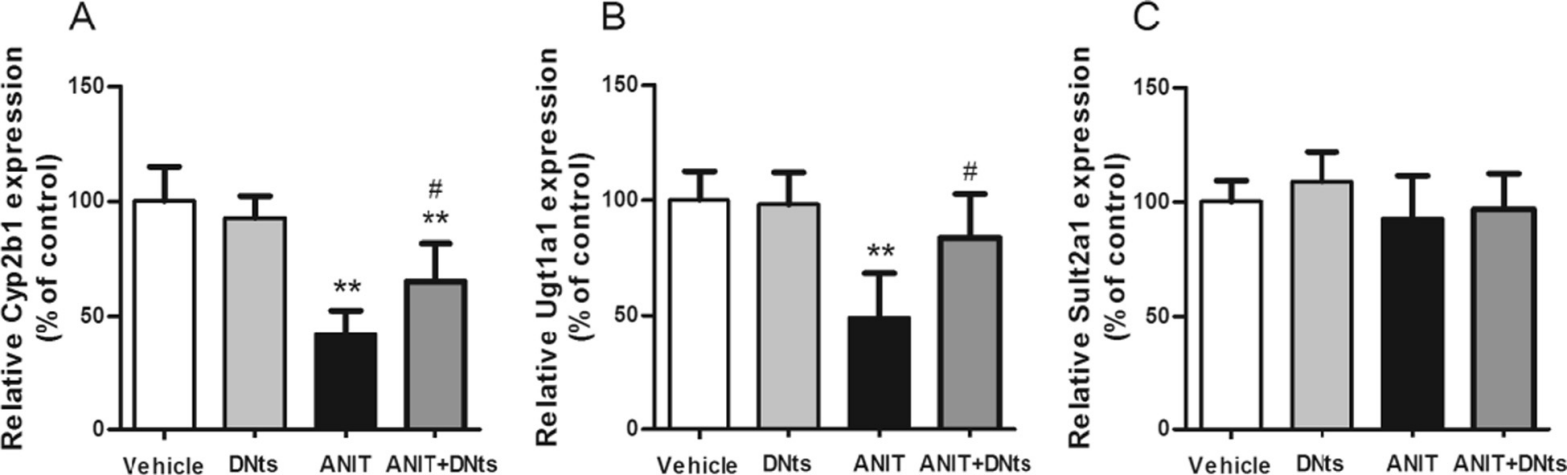
**Effects of DNts on the levels of Cyp2b1, Ugt1a1 and Sult2a1 in ANIT-induced cholestasis rats (n = 6 for each group).** Hepatic (A) Cyp2b1, (B) Ugt1a1 and (C) Sult2a1 levels were measured (n = 6 for each group). Values are expressed as folds of control. Each bar represents represents the mean ± SD of three independent experiments. Significant difference between two groups, *p < 0.05, **p < 0.01 vs. control group; ^#^*p* < 0.05, ^##^*p* < 0.01 vs. the ANIT-treated group.

The protein levels of endogenous metabolic enzymes in liver tissue microsomes was further confirmed. The protein levels of Cyp2b1 and Ugt1a1 in the model group were obviously reduced (Figure 8; *p* < 0.01). But the hepatic Sult2a1 level was not changed in ANIT-intoxicated rats (Figure 8). Whereas, DNts treatment notably reversed the decline of these metabolic enzymes except Sult2a1 compared with that in ANIT-treated rats (Figure 8; *p* < 0.01). Moreover, pretreatment of DNts alone did not affect the hepatic protein levels of Cyp2b1, Ugt1a1 and Sult2a1 (Figure 8).

## Discussion

The pretreatment of DNts significantly reversed ANIT-induced elevation of serum ALT, AST, ALP, T-Bil, D-Bil and TBA levels at 48h after ANIT treatment in consistent with previous research [23]. We presented here the evidence that DNts protected rats from the accumulation of bile acids in the liver and subsequent development of liver injury with cholestasis induced by ANIT, in association with an augmentation of bile acid and bilirubin excretion of bile and urine.

In the current study, we further investigated whether the protective effect of DNts against ANIT-induced cholestasis might be related to the expression of metabolic enzymes and transporters. The metabolic enzymes participate in critical processes including the biosynthesis of bile acids and the detoxification of accumulating biliary compounds. The hepatic metabolic enzymes may render hydrophobic substrates less toxic and better soluble for biliary and urinary excretion via phase I hydroxylation and phase II conjugation to counteract cholestatsis [24]. Our findings displayed that DNts pretreatment significantly up-regulated both mRNA and protein expressions of hepatic metabolic enzymes including Cyp2b1 and Ugt1a1 in ANIT-induced cholestasis rats. These results indicated that the up-regulation of mRNA and protein expressions of the phase I hydroxylation enzymes (Cyp2b1) and phase II conjugation enzymes (Ugt1a1) induced by DNts might counteract cholestatic liver damage by detoxification of accumulating biliary compounds.

Basolateral uptake transporters, such as Ntcp and Oatps, transport bile acids and organic anions from sinusoidal blood into hepatocytes [9-11]. In contrast, basolateral export transporters, such as Mrp3, Mrp4 and Ostα/β transport bilirubin glucuronide and bile acids, respectively, from hepatocytes into blood during cholestasis [10,25]. Bile acids transport at canalicular membrane of the hepatocyte is mediated by ATP-binding cassette transporters, including Bsep, Mrp2, and Mdr2 [26]. The export of toxic bile acids by canalicular transporters is probably an alternative escape route during intrahepatic cholestasis [8]. At 48 h after ANIT treatment, we found that spontaneous hepatic anticholestatic defenses which comprised down-regulation of import systems (e.g., Ntcp, Oatp1) and adaptive induction of basolateral alternative export pump (e.g.,Mrp3) as well as the canalicular transporters, such as MDR2 were formed in ANIT- intoxicanted rats. However, intrinsic hepatocellular adaptive induction of transporters in cholestasis is too weak to prevent ongoing liver injury [27]. Indeed, DNts pretreatment induced mRNA and protein expressions of hepatic canalicular export transporters Bsep and Mdr2, beyond what was seen following administration of ANIT alone. In addition, DNts reversed ANIT-induced down-regulation of mRNA and protein levels of Oatp1 and Ntcp for the attenuation of cholestasis has been performed.

Mrp2, which is another key canalicular transporter, belongs to ATP-binding cassette proteins localized to the canalicular domain of the hepatocyte. Recent studies have demonstrated that, Mrp2 undergoes endocytic retrieval from the canalicular membrane into an intracellular compartment in several models of cholestasis [20,28]. Our results showed that hepatic mRNA expression and protein levels of MRP2 did not changed in ANIT-treated rats. In contrast, Mrp2 in intracellular membranes was significantly increased in ANIT-intoxicated rats. The lack of change of Mrp2 content in mixed plasma membranes in response to ANIT may be tentatively explained by the fact that intracellular accumulation of Mrp2 in enriched microsomal membranes represents a small fraction of the total amount of transporter in the cell according to previous report [20]. It is possible that the protective effects of DNts were through other alternative mechanisms for the elevation of renal and biliary excretion of bile acid and bilirubin with unchanged expression of total Mrp2 in the kidney and liver.

Furthermore, Liver disease is often accompanied by renal dysfunction. After ANIT treatment, renal mRNA expressions of Mrp2, Mrp3, Mrp4 and Ostβ were significantly up-regulated. In contrast, renal mRNA level of Asbt was significantly down-regulated, accompanied by increased concentrations of bile acid and bilirubin in urine. These results indicated that adaptive responses to cholestasis also occur in the kidney in an effort to eliminate excess bile acids and toxic compounds from circulation which is according with previous report [29]. In addition, DNts pretreatment further increased the renal mRNA level of Ostβ in ANIT-intoxicated rats, accompanied by further increase in urinary excretion of bile acid and bilirubin (Figure 7).

## Conclusions

In summary, Danning tablets provides protection from ANIT-induced cholestasis with liver injury through adaptive responses in both the kidney and liver that enhance the expressions of hepatic canalicular efflux transporters (Bsep and Mdr2), renal efflux transporter Ostβ and bile acid-detoxifying enzyme (Cyp2b1 and Ugt1a1) and attenuate the hepatic Mrp2 translocation, accompanied by further increase in urinary and biliary excretion of bile acid and bilirubin.

## Abbreviations

ANIT: α-naphthylisothiocyanate; ALT: Alanine aminotransferase; AST: Aspartateaminotransferase; ALP: Alkaline phosphatase; TBIL: Total bilirubin; DBIL: Direct bilirubin; TBA: Total bile acid; DNts: Danning tablets; Cyp: Cytochrome P450; Ugt: Uridine diphosphate-5¢-glucuronosyl transferase; Sult: Sulfotransferases; Ntcp: Na^+^ taurocholate cotransporting polypeptide; Oatp: Organic anion-transporting polypeptide; Ost: Organic solute transporter; MRP: Multidrug resistance-associated protein; Bsep: Bile salt export pump; Mdr: Multidrug resistance protein; Asbt: Apical sodium dependent bile acid transporter; Gapdh: Glyceraldehyde-3-phosphate dehydrogenase; B.W.: Body weight.

## Competing interests

The authors declare that they have no competing interests with other people or organizations that can inappropriately influence their work. And there are no potential conflicts of interest including financial, employment, consultancies, stock ownership, honoraria, paid expert testimony, patent applications and registrations, and grants or other funding. In addition, there are no competing interests between Shanghai Hutchison Pharmaceuticals and Institute of Traditional Chinese Materia Medica, Shanghai University of Traditional Chinese Medicine.

## Authors’ contributions

ZW and LY, as the principal investigator, were responsible for the concept and design of the study. LD did the whole experiments of the study and wrote the manuscript. BZ and CZ conducted part of the experiments. All authors participated in the preparation of, and have approved the final version of the manuscript.

## Pre-publication history

The pre-publication history for this paper can be accessed here:

http://www.biomedcentral.com/1472-6882/14/249/prepub
